# Coumarin-chalcone hybrid instigates DNA damage by minor groove binding and stabilizes p53 through post translational modifications

**DOI:** 10.1038/srep45287

**Published:** 2017-03-28

**Authors:** Raghib Ashraf, Hamidullah, Mohammad Hasanain, Praveen Pandey, Mayank Maheshwari, L. Ravithej Singh, M. Quadir Siddiqui, Rituraj Konwar, Koneni V. Sashidhara, Jayanta Sarkar

**Affiliations:** 1 Biochemistry Division, CSIR-Central Drug Research Institute, Sector-10, Jankipuram Extension, Lucknow, 226 031, India; 2 Endocrinology Division, CSIR-Central Drug Research Institute, Sector-10, Jankipuram Extension, Lucknow, 226 031, India; 3 Medicinal and Process Chemistry Division, CSIR-Central Drug Research Institute, Sector-10, Jankipuram Extension, Lucknow, 226 031, India; 4 KS # 101, Tata Memorial Centre, Advanced Centre for Treatment, Research and Education in Cancer, Kharghar, Navi Mumbai, 410 210, India; 5 Academy of Scientific and Innovative Research, Chennai, 600113, India

## Abstract

S009-131, a coumarin-chalcone hybrid, had been shown to possess anti-proliferative and anti-tumour effect by triggering apoptosis. In this report, we investigated role of DNA damage signalling pathway in S009-131 induced cancer cell death. Here we show that S009-131 causes DNA damage by potential binding to the minor groove which led to the phosphorylation and activation of ATM and DNA-PK, but not ATR, at earlier time points in order to initiate repair process. S009-131 induced DNA damage response triggered activation of p53 through phosphorylation at its key residues. Pharmacological inhibition of PIKKs abrogated S009-131 induced phosphorylation of p53 at Ser 15. DNA damage induced phosphorylation resulted in reduced proteasomal degradation of p53 by disrupting p53-MDM2 interaction. Additionally, our docking studies revealed that S009-131 might also contribute to increased cellular p53 level by occupying p53 binding pocket of MDM2. Posttranslational modifications of p53 upon S009-131 treatment led to enhanced affinity of p53 towards responsive elements (p53-RE) in the promoter regions of target genes and increased transcriptional efficiency. Together, the results suggest that S009-131 cleaves DNA through minor groove binding and eventually activates PIKKs associated DNA damage response signalling to promote stabilization and enhanced transcriptional activity of p53 through posttranslational modifications at key residues.

DNA damage response is a coordinated reaction of a cell wherein an array of cellular proteins works in harmony to preserve genomic integrity upon an assault to the cellular DNA. In physiological context, response to DNA damage involves arrest of cell division cycle, up regulation of cellular deoxyribonucletide level, transactivation of genes involved in repair process and induction of apoptosis in case of irreparable lesions^1^. DNA damage can be correlated to the genesis of various human disorders including cancer which are caused as a result of erroneous repair process^2^. Paradoxically, it is also exploited by several chemotherapeutic agents to trigger cancer cell death. Numerous anticancer drugs such as cisplatin, 5-fluorouracil (5FU), camptothecin, etoposide etc as well as radiotherapy which are extensively used in the clinics rely on DNA damage to kill cancer cells. The MRN complex (comprising of Mre11, Rad 50 and NBS1) and replication protein A (RPA) are two major sensors of type specific DNA damages which activate key regulators of repair pathways ataxia telangiectasia mutated (ATM) and ataxia telangiectasia and Rad3 related (ATR) kinases, respectively. These two transducer kinases, in association with their respective partner proteins, subsequently phosphorylate Chk2 and Chk1 effector kinases and activate cell cycle checkpoints in order to repair DNA lesions before being replicated. Both transducer and effector kinases eventually activate several signalling molecules in transcription, DNA repair, cell cycle and apoptosis pathways^3^.

p53, commonly referred as “guardian of the genome”, is a tumour suppressor protein which is activated upon cellular stress and trigger cell cycle arrest, apoptosis, senescence and autophagy^4^,^5^. Nearly 50% of all cancer bear mutated p53 with altered activity while its function is impaired in rest of the human tumours owing to the deregulation of upstream pathways^6^. A very low level of DNA damage can be sensed by p53 pathway which in turn contributes to the repair process^4^ or promotes cell cycle arrest and apoptosis. DNA damage signalling causes induction of p53 through posttranslational modifications at several amino acid residues. Notable among them are phosphorylation at Ser 15, Ser 20, Ser 46, Ser 392 and Thr 18 which enhance its transcriptional efficiency and prevent proteasomal degradation by inhibiting interaction with mouse double minute 2 homologue (MDM2) protein^7^. The phosphorylation of p53 upon DNA damage is mediated by inducer [ATM, ATR and DNA-dependent protein kinase catalytic subunit (DNA-PK)] as well as effector (Chk1 and Chk2) kinases. Additionally, activated ATM, following DNA damage, phosphorylates MDM2 at serine 395 and thereby diminish its ability to degrade p53^8^. Activated p53 subsequently exert tumour suppressive activity through transcription dependent and independent manner.

Coumarin and chalcone are two important bioactive pharmacophores found in plants as secondary metabolites and possess diverse range of biological activities including therapeutic potential against cancer^9^,^10^. Pharmacological efficacy of these “active principles” depends on the pattern of substitution as well as conjugation with other moieties. In the course of design, synthesis and biological evaluation of a series of coumarin-chalcone hybrids, we identified a compound (S009-131) with promising *in vitro* anti-cancer activity against a panel of human cancer cell lines^11^. Our subsequent study revealed activation of intrinsic pathway of apoptosis by S009-131 which was mediated through induction of ROS and modulation in expression of Bcl2 family of proteins^12^. We also showed *in vivo* efficacy of this molecule in *nod* SCID mice bearing xenograft of human cervical carcinoma cells, HeLa^12^. While coumarin is primarily known to have protective effect towards damage to genomic DNA owing to its anti-oxidant potential^13^,^14^, it has also been demonstrated to bind DNA minor grooves by several biophysical techniques^15^. On the other hand, chalcone and its derivatives are shown to cause DNA strand breaks^16^,^17^,^18^. Our earlier report showed induction of p53 by the hybrid molecule (S009-131) during apoptosis in human cancer cells^12^. In the present study we demonstrate that induction of p53 was mediated through posttranslational modifications following activation of DNA damage signalling which further triggered downstream signalling pathways to induce cancer cell death.

## Results

### S009-131 induces DNA damage response

**Figure 1:**
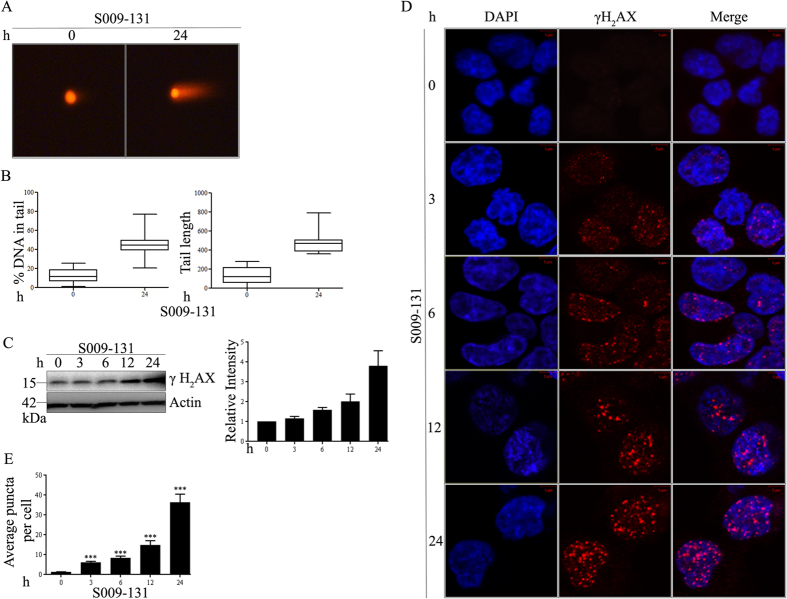
S009-131 induced DNA damage in HCT116 cells. (**A**) Cells were treated with the molecule at 7.5 μM for 24 h and comet assay was done to determine the extent of DNA damage. (**B**) Tail length and percentage of DNA in tail was measured (minimum 28 cells were analysed for each treatment condition) by Image J and represented with box-and-whisker plot. (**C**) HCT116 cells were incubated with S009-131 for different time period. Cell lysates were then subjected to immunoblotting using anti-γ-H2AX antibody and analysed densitometrically. (**D**) Representative confocal images to show the γ-H2AX puncta at different time intervals on S009-131 treatment. (**E**) Bar graph showing average puncta per cell at different time interval (minimum 24 cells were considered in each group).

In our previous work, we demonstrated a steady increase in p53 level during S009-131 induced apoptosis in C33A cells^12^. This was further confirmed in HCT 116 cells harbouring wild type p53 (described later in this section). Concurrently, the molecule caused accumulation of HCT116 cells at G2/M phase (Supp Fig. 1A) replicating previous reports^12^. The strong linkage of these two cellular events with biological consequences of DNA repair pathways raised the possibility that S009-131 might cause DNA damage. Indeed, we observed formation of DNA tail with higher percentage of DNA in tail region in S009-131 treated HCT116 cells in single cell neutral comet assay in comparison to untreated controls indicating induction of DNA damage by the molecule (Fig. 1A and B). This was additionally supported by the results of plasmid nicking assay (Supp Fig. 1B) where a steady increase in the intensity of bands representing open circular form (OC) and linear form (L) of plasmid was observed with increasing concentration of S009-131 suggesting DNA break^19^. Likewise, the compound caused slight shifting of the respective bands indicating overall conformational changes of the DNA. To further confirm DNA damage, HCT116 cells were treated with S009-131 for different time intervals at IC_50_ concentration (Supp Fig. 1C and D) and level of phosphorylated H2AX (γ-H2AX), a well-known marker of DNA damage, was measured by Western blot assay. As shown in Fig. 1C, incubation of cells with S009-131 resulted in increase of γ-H2AX level where highest expression was observed at 24 h. Formation of γ-H2AX specific foci in cell nuclei is another characteristic morphological feature of DNA damage. Here, we assessed level of γ-H2AX specific foci in HCT116 cells by fluorescence microscopy before and after treatment with the compound. As can be seen in Fig. 1D, time dependent accumulation of γ-H2AX foci was observed in S009-131 treated cells from 3 h and subsequent time points. The changes were statistically significant from 3 h onwards post-exposure to S009-131 compared to untreated controls (Fig. 1E). Together, above results suggest induction of DNA damage by S009-131.

### S009-131 binds to the minor groove to make DNA lesions

**Figure 2:**
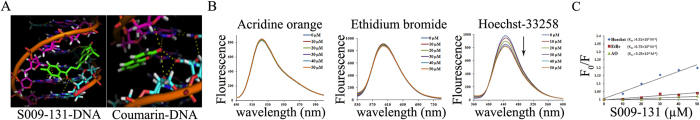
S009-131 binding to minor groove of DNA. (**A**) Comparative molecular docked structures of S009-131 and coumarin complexed with DNA showing specific minor groove binding. (**B**) Fluorescence spectral patterns for competitive displacement assay with different DNA interacting agents in presence of S009-131. (**C**) Stern-Volmer plot was sketched from fluorescence spectral data to calculate K_SV_ (Stern-Volmer constant) values.

DNA damage by genotoxic based chemotherapeutic agents can be induced either directly by physical interaction with genomic DNA or indirectly by inhibition of enzymes which are crucial in DNA replication and repair. Recently, physical binding of coumarin and its conjugates with DNA has been demonstrated by formation of complex between these pharmacophores and calf thymus DNA (ct-DNA)^15^,^20^. In line with these findings, our *in silico* studies indicated binding of S009-131 in minor groove of DNA possibly through hydrogen bonding with dG and dC nucleotide bases (Fig. 2A).

To get a better understanding on mode of S009-131-DNA interaction, we performed competitive displacement assay by exploiting fluorescence emission spectroscopy wherein acridine orange and ethidium bromide were used as representative DNA intercalating agents and Hoechst-33258 was used as standard minor groove binder. These dyes were added @ 5 μM concentrations to a solution of 50 μM ct-DNA to obtain saturated emission intensity followed by addition of test compound at increasing concentration. Ethidium bromide, acridine orange and Hoechst-33258 are fluorescent dyes which show negligible or weak emission in Tris-Hcl buffer in absence of nucleic acid due to the quenching by solvent molecules. But their emission spectra are greatly augmented when they form complex with DNA by their respective mode of binding. Replacement of dye by a competitive ligand from DNA-dye complex results in quenching of emission spectra and the changes in fluorescence intensity is easily interpreted^15^. As shown in Fig. 2B, gradual addition of S009-131 did not produce noticeable changes in emission spectra of AO-ct-DNA (acridine orange-ct-DNA) and Et-Br-ct-DNA (ethidium bromide-ct-DNA) adducts while the emission intensity of Hoechst-ct-DNA complex was significantly reduced in similar experiment with a K_sv_ value of 4.51 × 10^3^ M^−1^ much higher than K_sv_ values of other complexes that is 0.25 × 10^3^ M^−1^ and 0.75 × 10^3^ M^−1^ for AO-ct-DNA and Et-ct-DNA respectively (Fig. 2C) indicating displacement of Hoechst-33258 from minor groove. These findings suggest that S009-131 binds to minor grooves to initiate DNA damage.

### γ-H2AX response is activated by molecular sensors of DNA damage belonging to Phosphatidylinositol 3-kinase-related kinases (PIKK) family

**Figure 3:**
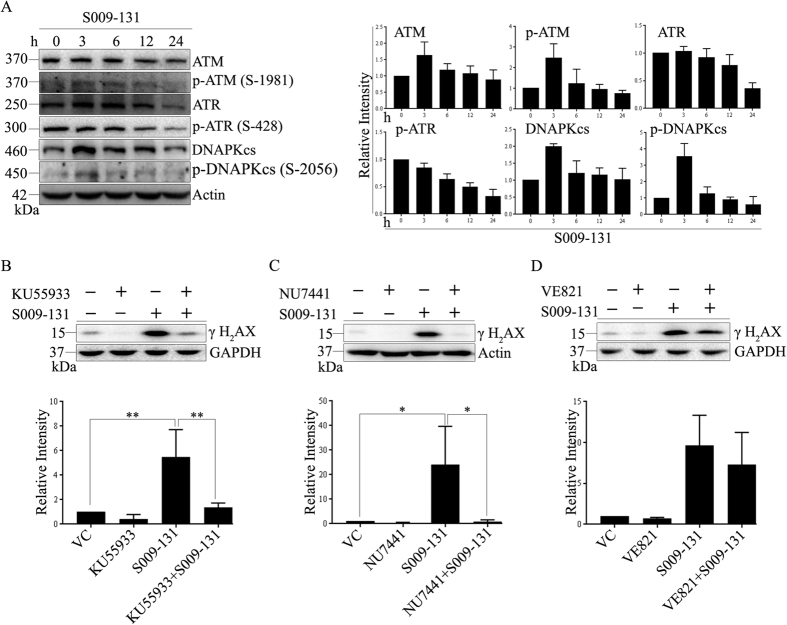
S009-131 induced DNA damage response through activation of PIKKs. (**A**) HCT116 cells were treated with 7.5 μM of S009-131 for indicated time points. Whole cell lysates were then collected and analysed by immunoblotting using antibodies specific to ATM, ATR and DNA-PK and their active phosphorylated form. (**B–D**) Cells were incubated with S009-131 with or without pre-treatment with specific PIKK inhibitors (KU5993, 10 μM; NU7441, 10 μM and VE821, 5 μM) and analysed for γ-H2AX expression by immunoblotting.

Members of PIKK family *viz.* ATM, ATR and DNA-PK are key regulators of DNA damage signals^21^. These kinases are recruited at DNA damage site depending upon type of lesion and mediate H2AX phosphorylation to trigger a cascade of downstream signalling including recruitment of repair factors, activation of cell cycle check point modulators and apoptosis. In the present study, activation of ATM, ATR and DNA-PK was monitored by studying their level of phosphorylation by Western blot assay before and after incubation with S009-131. As shown in Fig. 3A, S009-131 treatment resulted in increase in phosphorylated ATM and DNA-PK with highest level at initial time points of 3 h and 6 h which was reduced subsequently. Equally, S009-131 affected the expression of total ATM and DNA-PK in a similar pattern. On the contrary, level of phosphorylated ATR was steadily decreased upon S009-131 exposure which was in concordance with decrease in total ATR expression. To gain further insight, we used specific inhibitors KU55933 (for ATM), NU7441 (for DNA-PK) and VE-821 (for ATR) and monitored γ-H2AX level after S009-131 treatment by Western blot assay. As shown in Fig. 3B, phosphorylation of H2AX at Ser 139 was reduced upon pre-incubation of cells with KU55933. Similar results were obtained when cells were pre-treated with NU7441, and to a much lesser extent with VE-821 (Fig. 3C and D) suggesting involvement of mainly ATM and DNA-PK in phosphorylating H2AX.

### S009-131 stabilizes p53 through post translational modifications

**Figure 4:**
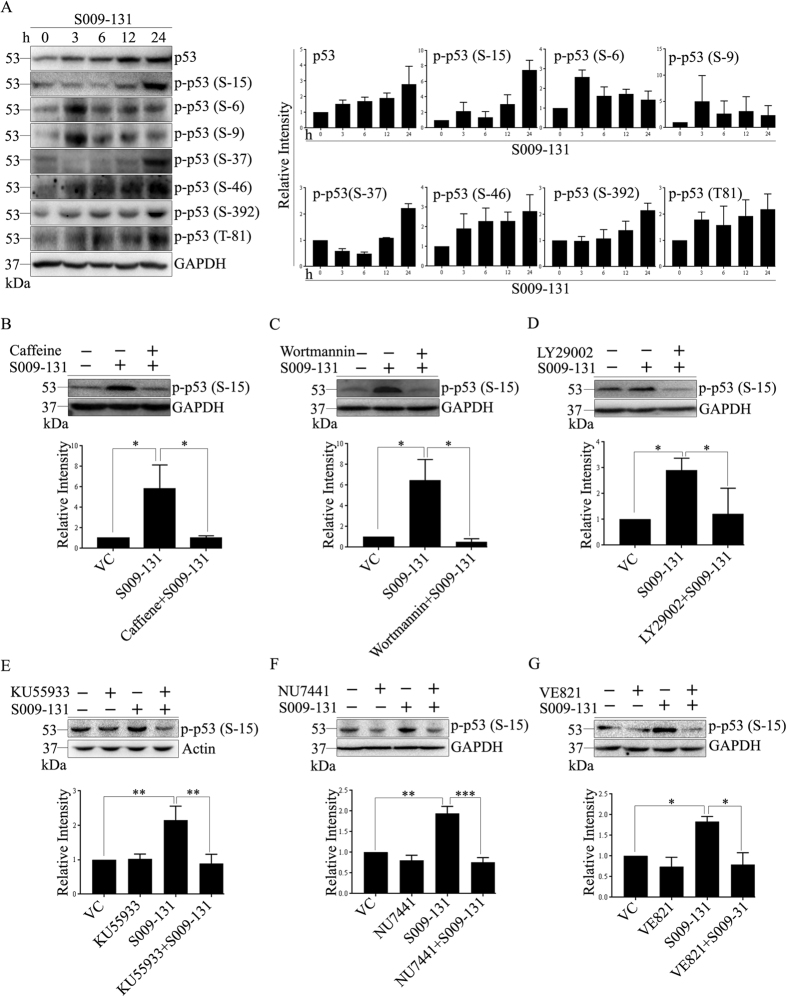
Effect of S009-131 on p53 phosphorylation. (**A**) HCT116 cells were treated with S009-131 for different time points. The protein lysates were then analysed by immunoblotting to detect phosphorylation state of p53 at indicated residues. (**B–D**) Influence of PIKKs on S009-131 induced p53 phosphorylation at Ser 15 was assessed by 2 h pre-incubation of cells with caffeine (1 mM), Wortmannin (10 μM), LY29002 (20 μM) and (**E–G**) with specific inhibitors.

Apart from H2AX, p53 is another effective target of PIKKs which is activated upon DNA damage and triggers apoptosis and cell cycle arrest. In consistency with our previous report^12^, we observed up regulation of p53 in HCT116 cells following S009-131 treatment (Fig. 4A). p53 is phosphorylated at key regulatory sites by ATM, ATR and DNA-PK which contributes to its stabilization and enhanced transcriptional efficiency^22^,^23^. In the present study, phosphorylation of p53 in HCT116 cells was assessed by Western blot assay using several phospho-p53 specific antibodies before and after exposure to S009-131. Our results demonstrate that S009-131 treatment caused transient phospophorylation of p53 at Ser 6 and Ser 9 at early stage (3 h) which reduced further in subsequent time points (Fig. 4A). Conversely, there was steady increase in p-p53 (Ser 392 and Ser 46) upon S009-131 treatment while phosphorylation at Ser 15 and Ser 37 appeared 12 h onwards (Fig. 4A). Level of p-p53 (Thr 81) was found to be enhanced in early as well as late time points (Fig. 4A). Since p-p53 (Ser 15) is widely considered as an indicator of p53 activation upon DNA damage, we further confirmed role of PIKKs in phosphorylating p53 at Ser 15 and initiation of DNA damage response pathway. Cells were pre-incubated with broadly active Phosphoinositide 3-kinase (PI3K) inhibitors wortmannin, caffeine and LY294002 before S009-131 treatment and level of p-p53 (Ser 15) was monitored by immunoblotting. While ATM and DNA-PK are more sensitive to wortmannin^24^,^25^, caffeine has relative specificity towards ATM and ATR^25^. On the other hand, LY294002 is comparatively more potent inhibitor of DNA-PK^26^. Here, pre-treatment of cells with these inhibitors diminished S009-131 induced phosphorylation of p53 (Ser 15) (Fig. 4B–D). To determine specifically whether ATM, ATR and DNA-PK were indeed regulating phosphorylation of p53, HCT116 cells were pre-incubated with specific inhibitors and level of p-p53 (Ser15) was measured at 24 h after S009-131 treatment. As shown in Fig. 4E, inhibition of ATM kinase by KU55933 resulted in marked reduction of p-p53 (Ser 15). Similar results were obtained when ATR and DNA-PK were down regulated with respective inhibitors (Fig. 4F and G).

**Figure 5:**
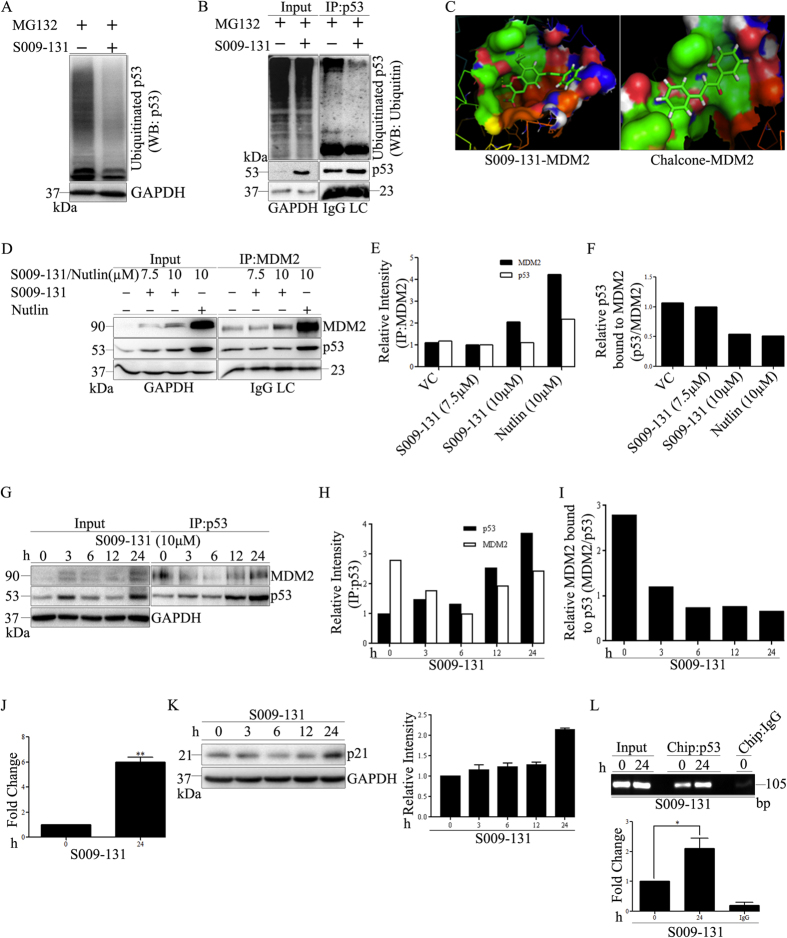
S009-131 protected p53 from proteasomal degradation and enhanced its transcriptional efficiency. (**A**) HCT116 cells were co-incubated with 10 μM MG132 and 7.5 μM S009-131. Whole cell lysates were analysed by immunoblotting with anti-p53 antibody. (**B**) Cell lysates were also subjected to immunoprecipitation (IP) using 1 μg of anti-p53 antibody followed by Western blot for p53 associated ubiquitins. (**C**) Predicted binding models for S009-131 and chalcone with MDM2. (**D**) HCT116 cells were treated with S009-131 and Nutlin at indicated concentrations for 24 h. Five hundred micrograms of protein lysates were immunoprecipitated with anti-MDM2 antibody and bound p53 was detected by immunoblotting. (**E** and **F**) Band intensities of Co-IP assay were analysed by Image J software and plotted graphically. (**G**) Western blot assay was performed to show the effect of S009-131 (10 μM) on p53-MDM2 interaction after immuonoprecipitating protein lysates with anti-p53 antibody. (**H** and **I**) Band intensities were determined by Image J software and represented graphically. (**J**) Binding affinity of p53 towards responsive elements (p53-RE) at promoter region before and after S009-131 treatment was determined by co-transfecting cells with EGFP-C1 and p53-Luc Cis Reporter plasmids. Luminescence were normalised by comparing with relative fluorescence in treated and untreated cells. (**K**) Western blot assay of whole cell lysates from S009-131 treated HCT116 cells to determine p21 protein level. (**L**) Chromatin immunoprecipitation (ChIP) assay was performed by immunoprecipitating chromatin complex from S009-131 treated and untreated cells with anti-p53 antibody followed by quantitative RT-PCR using p21 promoter specific primers.

DNA damage response induced phosphorylation plays an essential role for stabilization of p53 by preventing proteasomal degradation. Here, we assessed differential ubiquitination of p53 in HCT116 cells by co-incubating 10 μM of MG 132 with or without S009-131 for 24 h and cell lysates were subjected to immunoblotting using anti-p53 antibody as well as immunoprecipitation with p53 followed by immunoblotting with anti-ubiquitin antibody. S009-131 treatment resulted in considerable up regulation of p53 which was associated with marked reduction of ubiquitination with reduced smearing pattern (Fig. 5A and B input). Likewise, less amount of ubiquitin was co-immunoprecipitated with p53 in S009-131 treated cells compared to untreated control (Fig. 5B).

### S009-131 inhibits binding of p53 with MDM2

MDM2 is an E3 ligase which negatively regulates p53 by interacting with it and catalysing polyubiquitination to promote proteasomal degradation. Association of p53 with MDM2 is highly dependent on phosphorylation status of p53 which negatively regulates their interaction. In order to determine whether reduced level of p53 ubiquitination upon S009-131 treatment is due to decreased association between p53 and MDM2, HCT116 cell lysates were immunoprecipitated with anti-MDM2 antibody and level of associated p53 was detected by western blot assay. As can be seen in Fig. 5D–F, decreased amount of p53 was detected in MDM2 precipitates in S009-131 treated cells despite strong upregulation of p53 (Fig. 5D, input). Similar effects were noticed in reverse co-immunopreciptation assay where the results were validated by immublotting of MDM2 in p53 immunoprecipitates (Fig. 5G–I). Temporal activation of p53 by small molecules can occur by blocking p53-MDM2 interaction through occupying p53 binding pocket of MDM2. To this end, docking study was performed to explore possible interaction of S009-131 with MDM2. As shown in Fig. 5C, S009-131 lodged into the p53 binding pocket of MDM2 with the possibility of hydrogen bonding with Leu 54 and Gln 18 residues which is in concordance with previous reports on MDM2-chalcone interaction^27^. Interestingly, the relative binding energy for S009-131-MDM2 complex was −297 kJ/mol compared to −209 kJ/mol for chalcone-MDM2 complex implying higher binding potential of the hybrid molecule for MDM2.

### S009-131 enhances transcriptional efficiency of p53

Phosphorylation of p53 at different sites facilitates its promoter binding affinity either through enhanced sequence specific DNA binding or by interaction with key transcriptional regulatory proteins. In the present study, we found an increase in phospho-p53 (Ser 46 and Ser 392) (Fig. 4A) which is believed primarily to control transcription function of the tumour suppressor^28^. To confirm increase in p53 transactivation potential, we performed luciferase reporter assay by transfecting HCT 116 cells with plasmids harbouring p53 responsive elements at promoter region. As shown in Fig. 5J, S009-131 exposure resulted in significantly increased transactivation ability of p53 responsive promoter compared to untreated control. Accordingly, expression of p21 gene, a well known target of p53 involved in cell cycle arrest was found to be elevated upon S009-131 exposure (Fig. 5K). To further validate our results, enhanced recruitment of p53 to chromatin was demonstrated by ChIP assay using p21 promoter specific primers. As can be seen in Fig. 5L, significantly high copy number of p21 promoter specific amplicon was observed in p53 precipitates in compound treated cells compared to untreated control suggesting more recruitment of p53 to p21 promoter in response to S009-131 treatment.

## Discussion

Although S009-131 has been shown to trigger apoptosis in human cancer cells^12^, the underlying mechanisms remain obscure. DNA damage response signalling is one of the crucial pathways which culminates in inducing apoptosis and has been exploited by several chemotherapeutic drugs to exert anticancer activity^29^. In the course of further investigation of S009-131 induced apoptosis, we observed induction of DNA damage by this hybrid molecule as demonstrated by formation of DNA tails in comet assay. Being a small molecule of molecular weight 421, it is quite possible that S009-131 interacts with genomic DNA through any of the three mechanismsa) intercalation, b) groove binding and c) covalent binding to cause DNA damage^30^. In a recent report, coumarin has been shown to form complex with DNA *in vitro* by binding to the minor groove^15^. Consistent with this observation, we also found interaction of S009-131 with calf thymus DNA through binding to the minor groove which was supported by bioinformatics analysis as well. Therefore, it is probable that the coumarin moiety of the hybrid molecule might be responsible for binding to the cellular DNA leading to its damage. It is also likely that coumarin mediated binding of S009-131 to DNA minor groove additionally facilitates cleavage of DNA by chalcone pharmacophore^17^. However, the possibility of other indirect modes of DNA cleavage, most likely through enhanced ROS generation, by S009-131 cannot be ruled out^12^.

In human, DNA damage response is essentially regulated by ATM, ATR and DNA-PK kinases that initiate repair process by activating downstream signalling cascades. Activated ATM, ATR and DNA-PK phosphorylate H2AX at damage sites which serves as a platform for recruitment of chromatin remodelling factors and repair proteins. In the present study, S009-131 triggered H2AX phosphorylation at 12 h and 24 h post treatment as well as caused steady increase in γ-H2AX specific foci in HCT116 nuclei suggesting activation of DNA damage response pathway. Likewise, the compound induced phosphorylation of ATM (Ser 1981) and DNA-PK (Ser 2056) at earlier time points (which declined later) implying transient activation of these PIKKs by the molecule. On the contrary, exposure of HCT 116 cells to S009-131 resulted in time dependent depletion of p-ATR (Ser 428) and total ATR protein. This could be due to the reduction of half-life of ATR because of impairment of binding of ATR to hsp90 by coumarin moiety of the molecule^31^,^32^. Given that phosphorylation of PIKKs is critical in DNA damage repair process, it is possible that ATM and DNA-PK are two crucial players in amplifying the S009-131 induced damage signal. Their relevance in S009-131 induced signalling was additionally confirmed by using specific inhibitors which diminished γ-H2AX and p-p53 (Ser 15) level. Nevertheless, pharmacological inhibition of ATR also caused reduction in γ-H2AX and p53 phosphorylation implying involvement of ATR as well in execution of S009-131 induced signalling. Importantly, reduction in γ-H2AX was highest when DNA-PK specific inhibitor NU7441 was used; making us to believe that DNA-PK might be playing major role in generating early signals for DNA damage. Since none of the inhibitors could completely abolish S009-131 mediated phosphorylation of p53 (Ser 15) and H2AX (Ser 139), our results further reiterate the perception of functional redundancy among PIKKs in transducing DNA damage signal^21^.

p53 remains at the hub of DNA damage induced signalling cascade. DNA damage mobilizes p53 function to define biological outcomes like repair, cessation of cell growth and promotion of cell death depending upon type and intensity of the lesion^22^ where excessive DNA lesions upregulate p53 expression to trigger apoptosis^33^. In line with this notion, we found enhanced expression of p53 in S009-131 treated HCT116 cells. Upregulation of p53, upon DNA damage, is due to reduced degradation instead of augmented transcription or translation^33^. Under normal physiological condition, p53 is a short lived protein with a half-life of 10-20 min in most cells. Intracellular p53 homeostasis is tightly controlled through ubiquitination by MDM2 ubiquitin ligase and subsequent proteasomal degradation. Conversely, MDM2 is transactivated by p53 through auto regulatory loop. Posttranslational modifications of p53 at critical sites disrupt the interaction and protect p53 from proteasomal degradation. Better characterized among such alterations are phosphorylation of p53 at Ser 15 and Ser 37^34^. In the present study, S009-131 triggered phosphorylation of p53 at Ser 15 and Ser 37 implying reduced binding of p53 with MDM2 which was further demonstrated by co-immunoprecipitation assay. Alternatively, several small molecules activate p53 through disruption of p53-MDM2 interaction by occupying p53 binding pocket. In our docking studies, we saw reasonable fitting of S009-131 to the MDM2 binding pocket which might be facilitated by chalcone moiety of the hybrid^35^. Collectively, our results suggest that S009-131 upregulates p53 by impairing MDM2 mediated proteasomal degradation.

To exert tumour suppressive effect, p53 primarily relies on transcriptional regulation of a large subset of genes in apoptosis pathway. Posttranslational modifications of p53 at specific residues play decisive role in promoter selectivity and transcriptional efficiency^36^. Phopsphorylation of p53 at Ser 15 is the primary site of modification which is catalysed by ATM, ATR and DNA-PK in response to DNA damage^37^,^38^. Ser 15 phosphorylation serves as a platform for subsequent series of posttranslational modifications including acetylation at multiple lysine residues and thus facilitates p53 stabilization. Similarly, phosphorylation of p53 at multiple residues (Serines 9, 20 & 46 and Thr 18) is dependent on the prior phosphorylation at Ser 15^39^,^40^,^41^,^42^,^43^. In our study, S009-131 triggered phosphorylation of p53 at Serines 15, 37, 46, 81 and 392. Likewise, there was also robust induction in p53 phosphorylation at Ser 6 and Ser 9 at earlier time points upon S009-131 treatment which subsided later. Although the functional consequences of p53 phosphorylation at Ser 6 and Ser 9 has yet not been established, our results support the notion that phosphorylation of p53 at Ser 6 is one of the earliest DNA damage induced events and it bears a relationship with Ser 9 through similarity in time course of the phosphorylation^44^. Phosphorylation at Ser 46, -81 and -392 Serines 46, 81 and 392 activates p53’s function as transcription factor. In the present study, increase in p53 transcriptional efficiency was confirmed by luciferase reporter assay. Our study also revealed enhanced recruitment of p53 to the promoter regions of p21 by ChIP assay. Phosphorylation of p53 at Thr 81 by JNK contributes stabilization and increased transcriptional activity of p53^45^. Likewise, p53 phosphorylation at Ser 46 offers selectivity to transcribe proapoptotic genes^46^. While Ser 392 phosphorylation facilitates DNA binding by promoting oligomerization^47^, DNA damage induced phosphorylation at Ser 37 offers transcriptional activation of p53 by regulating sequence specific DNA binding^42^. Consistent with this notion, we observed higher binding affinity of p53 to responsive elements (p53-RE) upon S009-131 treatment. Equally, p-p53 (Ser 37) also contributes to p53 stability by impairing interaction with MDM2^42^. Taken together, our data indicate that S009-131 enhances transcriptional activity of upregulated p53 by promoting phosphorylation at multiple residues. However, given that S009-131 induces apoptosis in p53 deficient HCT116 (data not shown) and HeLa^12^ cells, it is possible that the anticancer activity of the molecule is not exclusively dependent on p53. As an alternative and similar to other DNA damage inducers, it may induce cell death by triggering p53 homologues p63 and/or p73, together with or independent of functional p53^48^,^49^.

Collectively, our findings highlight DNA damage response signalling as one of the likely mechanisms underlying S009-131 induced apoptosis. The compound cleaved DNA possibly through minor groove binding and activated PIKKs and downstream signalling cascades to promote posttranslational modifications of p53. S009-131 induced phosphorylation of p53 at key residues contributed to stabilization of the tumor suppressor by impairing MDM2 mediated proteosomal degradation. We also found that S009-131 triggered promoter binding efficacy and transcriptional activity of p53 by promoting phosphorylation at multiple residues.

## Material and Methods

### Chemicals and Antibodies

PhosSTOP was purchased from Roche Diagnostics (Indianapolis, Indiana, United States). Protein A & protein G agarose beads, ethanol, VE821 and mouse anti-phospho-H2AX (S139) (05–636, Lot #2310355) were purchased from Merck Millipore (Bedford, Massachusetts, USA). Clarity Western ECL substrate, 30% acrylamide/bis-acrylamide solution and non-fat dry milk were obtained from Bio-Rad (Hercules, California, USA). Pierce BCA protein assay kit, Pierce Magnetic ChiP Kit and M-PER reagent were obtained from Thermo Scientific (Rockford, Illinois, USA). NU7441, KU55933, rabbit anti-p53 (sc-6243, Lot #A1212), mouse anti-p53 (sc-126, Lot #F1113), mouse anti-MDM2 (sc-965, Lot #E0813), mouse anti-ATM (sc-23921, Lot #F2113), goat anti-ATR (sc-1887, Lot #E1613), mouse anti-DNA-PKcs (sc-5282, Lot #H0613) and mouse anti-p21 (sc-6246, Lot #I0111) antibodies were acquired from Santa Cruz Biotechnology (Santa Cruz, California, USA). HRP-conjugated anti-light chain specific antibodies (mouse and rabbit) were obtained from Jackson Laboratories (Bar Harbor, Maine, USA). Protein G & protein A conjugated magnetic beads were obtained from Invitrogen Corp (Carlsbad, California, USA). Prolong gold antifade reagent, Alexa Flour 594-conjugated secondary antibody, Lipofectamine 2000 and heat inactivated fetal bovine serum were purchased from Life Technologies (Carlsbad, California, USA). Mouse anti-GAPDH antibody (10–10011, Lot #0701370113 H) was purchased from ABGENEX Pvt. Ltd. (Bhubaneswar, Odisha, India). Rabbit anti-ubiquitin (3933, Lot #4), rabbit anti-phospho-Histone H2A.X (Ser 139) (2557, Lot #8), rabbit anti-phospho-DNA-PK (Ser 2056) (4215, Lot #1), rabbit anti-phospho-ATR (Ser 428) (2853, Lot #5), rabbit anti-phospho-p53 (Ser 6) (9285, Lot #5), rabbit anti-phospho-p53 (Ser 9) (9288, Lot #2), rabbit anti-phospho-p53 (Ser 15) (9284, Lot #15), rabbit anti-phospho-p53 (Ser 37) (9289, Lot #4), rabbit anti-phospho-p53 (Ser 46) (2521, Lot #5), rabbit anti-phospho-p53 (Thr 81) (2676, Lot #3) and rabbit anti- phospho-p53 (Ser 392) (9281, Lot #4) specific antibodies were purchased from Cell Signaling Technology (Danvers, Massachusetts, USA). Mouse anti-phospho-ATM (Ser 1981) (200-301-400, Lot #27452) antibody was obtained from Rockland Immunochemicals Inc (Limerick, Pennsylvania, USA). Mouse anti-β-Actin antibody (A2228), Nutlin-3 (N6287), Acridine orange (A6014), Ethidium bromide (E7637), Hoechst-33258 (94403) and the remaining biochemical/kits were obtained from Sigma-Aldrich (St. Louis, Missouri, USA).

### Cell culture

HCT116 wild type (p53 parental) and p53-/- cell lines were kind gift from Prof Bert Vogelstein (Johns Hopkins University) and were cultured in McCoy’s 5 A media supplemented with 10% fetal bovine serum at 37 °C with 5% CO_2_. Early passage cells (within passage 15) were included in experiments. Cell line was authenticated by STR profiling. Treatment of cells was carried out in growth medium at 37 °C at indicated time points.

### Sample preparation

A 10 mM stock solution of S009-131 was prepared by dissolving the compound in dimethyl sulfoxide (DMSO). Calf thymus DNA (ct-DNA) was prepared as 200 μM stock solutions by dissolving lyophilized ct-DNA in 10 mM Tris-HCl buffer (pH 7.2). The molarity of solution was calculated by exploiting mean extinction coefficient value of 6600 M^−1^ cm^−1^ for a single nucleotide at 260 nm^50^.

### SRB (Sulforhodamine B) assay

SRB assay for determining growth inhibitory activity of S009-131 was performed as described earlier^51^. Briefly, 10^4^ cells were grown overnight in each well of a 96-well tissue culture plate. Cells were then exposed to serial two fold dilutions of S009-131 for 48 h followed by fixation with ice-cold 50% Trichloroacetic acid (TCA) and staining with 0.4% (w/v) SRB in 1% acetic acid. Plates were subsequently washed and air dried. One hundred and fifty microliter of 10 mM Tris base was added to each well to dissolve bound dyes and plates were read colorimetrically at 510 nm absorbance.

### Luciferase assay

p53-Luc Cis Reporter plasmid was acquired from Agilent Technologies (Santa Clara, California, USA) and pEGFP-C1 plasmid from Clontech (Mountain View, California, USA). For Luciferase assay, 0.3 × 10^6^ cells were seeded in 6 well plate and grown for overnight. Next day, cells were subjected to serum starvation for 2 h in antibiotic free media and transfected with 2.5 μg of total plasmid (1.25 μg each of p53-Luc Cis Reporter and pEGFP-C1) using Lipofectamine 2000 according to manufacturer’s protocol. After 6 h of transfection, cells were supplemented with fresh complete media, incubated overnight and exposed to S009-131 for additional 24 h. Cells were then harvested with lysis buffer (1 M Tris, pH 8.0, 10% Triton X100) and luminescence was taken in GloMax 96 microplateluminometer (Promega, Madison, USA) after adding substrate (1 M KH_2_PO_4_, 0.5 M MgCl_2_, 0.1 M ATP, 10 mM luciferin disodium salt). Concurrently, equal amount of protein was taken to measure fluorescence at 485 nm excitation and 520 nm emission.

### Immunoblotting and immunoprecipitation

Treated and untreated cells were washed with ice-cold PBS and harvested for 30–40 min on ice using M-PER lysis buffer supplemented with protease and phosphatase inhibitors. Cell lysates were then centrifuged at 12000 g for 10 min and protein concentration of the supernatant was determined by Pierce BCA assay kit. For immunoblotting, 20–40 μg of protein was resolved in SDS-PAGE and probed with respective antibodies after transferring onto PVDF membrane. After incubation with appropriate secondary antibody, bands were detected with an enhanced chemiluminescence reagent and imaged by Chemidoc XRS^+^ Imaging system (Bio-Rad, California, USA). For immunoprecipitation, 500 μg of protein was diluted @ 1 mg/ml in M-PER reagent supplemented with protease inhibitors from which 60 μl was taken out as input. Remaining lysate was subjected to immunoprecipitation by overnight incubation at 4 °C with respective antibody on a rotating platform. Concurrently, 20 μl of protein A and G agarose beads were added to the lysate and incubated for 2 h. Beads were then washed three times with lysis buffer and boiled for 5 min with 2x loading dye before loading into SDA-PAGE. Densitometric analysis was done by Image J (NIH, Maryland, USA) software and depicted as relative intensity by normalizing with actin or GAPDH.

### Chromatin Immunoprecipitation (ChIP)

Chromatin immunoprecipitation was done using Pierce Magnetic ChIP Kit as per manufacturer’s protocol with minor modifications. Briefly, equal numbers (2 × 10^6^) of treated and untreated cells were cross linked with formaldehyde at 1% final concentration, washed with cold PBS and harvested in membrane extraction buffer supplemented with protease/phosphatase inhibitors. Cell nuclei were then re-suspended in MNase digestion buffer, incubated with MNase @ 0.005 U/μl and sonicated for 5 cycles with 10 s on and 30 s off. Digested chromatin was incubated overnight with anti-p53 (FL-393) antibody followed by addition of Protein A and G conjugated magnetic beads. After incubation for 2 h at 4 °C, protein-chromatin complex was recovered and 3 μl of eluted DNA was subjected to real time PCR using p21 promoter specific primers (forward 5′-GTGGCTCTGATTGGCTTTCTG-3′ and reverse 5′-AGGGCTTCCTCTTGGAGAA-3′^52^). Agarose gel images were acquired on Geldoc XR + Imaging system (Bio-Rad, California, USA).

### Cell cycle analysis

Effect of S009-131 on cell division cycle was checked in time dependent manner after staining cells with propidium iodide (PI). Summarily, 8 × 10^5^ HCT116 cells were seeded in T-25 flasks, grown overnight at 37 °C in 5% CO2 concentration and subsequently treated with S009-131 at indicated time points. Treated cells were acquired, fixed with cold ethanol and stained with PI (20 mg/ml). Cells were then analysed by FACS calibur flow cytometer (BD Biosciences, California, USA) employing “Cell Quest” software.

### Ubiquitination assay

*In vivo* ubiquitination of p53 was studied in HCT116 cells. Vehicle and S009-131 treated cells were co-incubated with MG-132 for 24 h, lysed with M-PER reagent and protein concentration was determined by BCA assay kit. Five hundred microgram of lysate was subjected to overnight immunoprecipitation using anti-p53 antibody (FL-393) followed by incubation with protein A and G agarose beads. Samples were then resolved in SDS-PAGE and transferred on nitrocellulose membrane. Membrane bound proteins were denatured with denaturing solution (6 M Guanidine-HCl, 20 mM Tris-HCl pH 7.5, 5 mM β-mercaptoethanol, 1 mM PMSF) to improve the exposure of ubiquitin epitopes, blocked with 5% milk in TBST and probed with anti-ubiquitin antibody. Positive signals were detected with an enhanced chemiluminescence reagent and imaged by Chemidoc XRS^+^ Imaging system.

### Comet assay

The neutral comet assay was carried out as described previously^53^. Briefly, glass slides were coated with 1% low melting agarose. Subsequently, a mixture of 3000 cells in 1% low melting agarose was overlaid onto the slide which was subsequently immersed into the lysis buffer (2% Sodium lauroylsarcosinate, 0.5 M Na_2_EDTA, 0.5 mg/ml proteinase K pH 8.0). After overnight incubation, electrophoresis was performed in a horizontal gel electrophoresis tank filled with fresh electrophoresis solution (90 mM Tris buffer, 90 mM boric acid, 2 mM Na_2_EDTA) at 20 V for 25 min. Slides were then briefly rinsed with electrophoresis buffer, stained with propidium iodide and observed under Nikon Eclipse TiE inverted microscope. Individual tail length as well as percentage of DNA in each tail was calculated by Image J software using automatic “open comet” plugins.

### Immunofluorescence

Immunofluorescence assay was performed as described previously^54^. Cells were grown overnight on coverslips, treated with S009-131 for indicated time points and fixed with 4% paraformaldehyde. After a brief wash with PBS, cells were permeabilized with 0.5% Triton X100 in PBS and blocked with 2% BSA for 1 h at RT. Cover slips were then incubated overnight with anti-phospho-Histone (S139) (05-636) antibody at 4 °C followed by 1 h incubation with fluorescence conjugated secondary antibody at RT. After three washes with PBS, coverslips were mounted on glass slides using ProLong Gold Antifade reagent containing DAPI. Slides were then examined under Carl Zeiss LSM 510 META confocal microscope (Jena, Germany) equipped with a Plan Apochromat 63x oil/1.4 NA DIC objective and γ-H2AX specific foci were counted by ImageJ software.

### Plasmid nicking assay

This assay was performed by incubating pCDNA3.1 plasmid (0.5 μg) with varying concentration of S009-131 (0 μM, 100 μM, 150 μM and 200 μM) in 25 μl of 10 mM Tris-HCl buffer (pH 7.4) at 37 °C for 24 hour. The plasmids were subsequently resolved on 1% agarose gel at 100 V for ~30 min with 0.5 μg/ml of EtBr and gel images were acquired on Geldoc XR + Imaging system.

### Fluorescence studies

The fluorescence emission spectra for displacement study was performed using standard DNA binding fluorophores, *i.e.* ethidium bromide (EB), acridine orange (AO) as standard intercalators and Hoechst-33258 as standard minor groove binder, in presence and absence of S009-131. In brief, 3 different sets of solutions containing 50 μM of ct-DNA and 5 μM each of the DNA binding dyes in buffer containing 10 mM Tris-HCl, pH 7.2 were incubated for 30 min. Subsequently, increasing concentrations of S009-131 (0–50 μM) was added to each of these solutions and alterations in fluorescence spectra was monitored on LS 50B Fluorescence/Luminescence Spectrometer with the use of High precision cell (Light Path: 10 × 2 mm) (PerkinElmer, Inc., Massachusetts, United States) at room temperature. EB-ct-DNA complex was excited at 476 nm and emission was recorded from 530 to 700 nm. Similarly, AO-ct-DNA complex was exited at 480 nm and Hoechst-ct-DNA was exited at 343 nm and their emission spectra were taken within the range of 490 to 600 nm and 360 to 600 nm, respectively. All the titrations were done in total of 700 μl buffer and suitable blanks were included in the experiment to nullify any noise.

### Docking Studies

HEX 8.0.0 software with an interactive molecular graphics tool was used for docking studies. It uses spherical polar Fourier (SPF) correlation approach to capture docking mode proteins and DNA with small molecule. The crystal structures of MDM2 (PDB ID code 4ZYF) and synthetic DNA dodecamer (PDB ID code 1BNA) were availed from the protein data bank (http://www.rcsb.org./pdb). Ligand three dimensional conformations were created by PRODRUG server with energy minimization advancement. Both the ligand and the receptor were put in the PDB format by using PyMol molecular graphics software. Prior to docking, all hetero atoms were removed from both ligand and receptor. The parameters used for docking was set as: correlation type – shape only; FFT mode – 3D; grid dimension – 0.6; receptor range – 180; ligand range – 180; twist range – 360; and distance range – 40. PyMol molecular graphic Software was used for the visualization of docked poses.

### Statistical analysis

Statistical analysis and graphs were made by GraphPad Prism7 software. All the results were depicted in the form of standard error mean. Significance of results were evaluated by two tailed t-test between two groups whereas 1-way ANOVA (Dunnett’s Multiple Comparison Test) done for multiple groups. *P* value less than 0.05 was considered significant.

## Additional Information

**How to cite this article:** Ashraf, R. *et al*. Coumarin-chalcone hybrid instigates DNA damage by minor groove binding and stabilizes p53 through post translational modifications. *Sci. Rep.* **7**, 45287; doi: 10.1038/srep45287 (2017).

**Publisher's note:** Springer Nature remains neutral with regard to jurisdictional claims in published maps and institutional affiliations.

## Supplementary Material

Supplementary Information
