# Supplementary material for: Coumarin-chalcone hybrid instigates DNA damage by minor groove binding and stabilizes p53 through post translational modifications
Source: Sci Rep. 2017 Mar 28;7:45287. doi: 10.1038/srep45287 (PMC5368660; doi:10.1038/srep45287)
Supplement: Supplementary Information [file srep45287-s1.pdf]

Coumarin-chalcone hybrid instigates DNA damage by minor groove binding and stabilizes p53 through post translational modifications

Raghib Ashraf[1], Hamidullah[2], Mohammad Hasanain[1], Praveen Pandey[1], Mayank Maheshwari[1], L. Ravithej Singh[3], M. Quadir Siddiqui[4], Rituraj Konwar[2,5], Koneni V. Sashidhara[3,5], Jayanta Sarkar[1,5]*

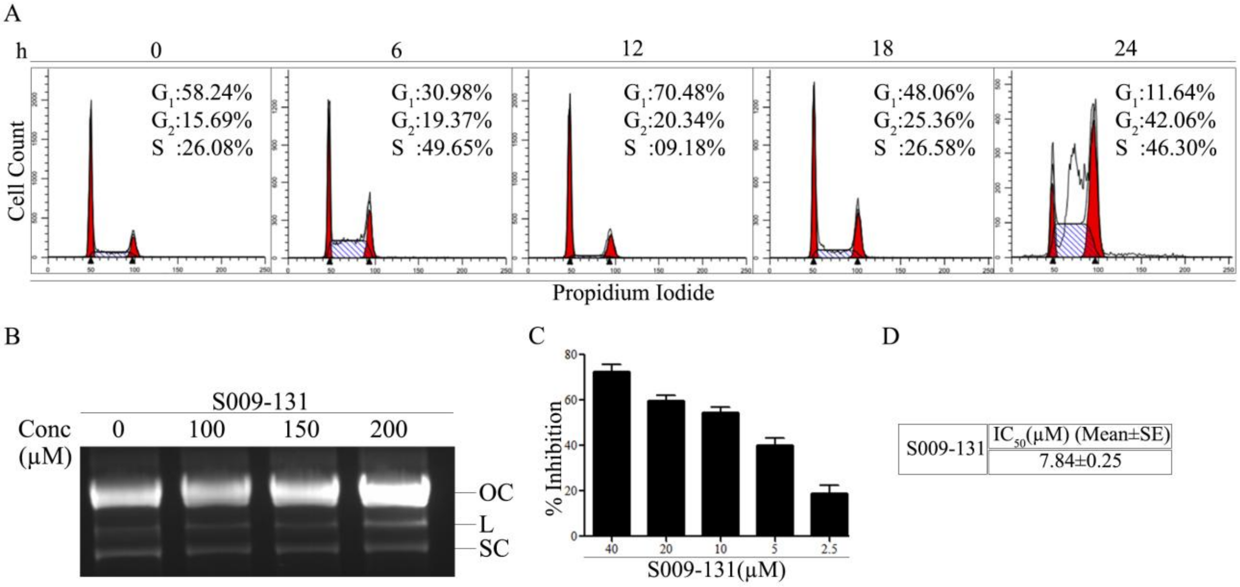

Supplementary Figure 1. *(A)* Effect of S009-131 on cell division cycle was determined by flow cytometry. Cells were incubated with the molecule at 7.5 μM concentration for indicated time points and cell cycle analysis was done after PI staining. *(B)* Plasmid nicking assay was performed by incubating pCDNA3.1 (0.5μg) with varying concentration of S009-131 as indicated for 24 h followed by agarose gel electrophoresis. *(C)* Anti-proliferative activity of S009-131 on HCT 116 cells. Cells were exposed to the compound for 24h at indicated concentration and % cell growth compared to untreated control was determined by SRB assay. *(D)* IC_{50} value of S009-131 as determined by dose response curve fitting.
